# Solvothermal‐Derived S‐Doped Graphene as an Anode Material for Sodium‐Ion Batteries

**DOI:** 10.1002/advs.201700880

**Published:** 2018-02-14

**Authors:** Bo Quan, Aihua Jin, Seung‐Ho Yu, Seok Mun Kang, Juwon Jeong, Héctor D. Abruña, Longyi Jin, Yuanzhe Piao, Yung‐Eun Sung

**Affiliations:** ^1^ Department of Chemistry MOE Key Laboratory of Natural Resources of the Changbai Mountain & Functional Molecules Yanbian University Yanji 133002 P. R. China; ^2^ Center for Nanoparticle Research Institute for Basic Science (IBS) School of Chemical and Biological Engineering Seoul National University Seoul 08826 Republic of Korea; ^3^ Department of Chemistry and Chemical Biology Cornell University Ithaca NY 14853 USA; ^4^ Graduate School of Convergence Science and Technology Seoul National University Seoul 08826 Republic of Korea

**Keywords:** anodes, graphene, sodium‐ion batteries, solvothermal methods, sulfur doping

## Abstract

Sodium‐ion batteries (SIBs) have attracted enormous attention in recent years due to the high abundance and low cost of sodium. However, in contrast to lithium‐ion batteries, conventional graphite is unsuitable for SIB anodes because it is much more difficult to intercolate the larger Na ions into graphite layers. Therefore, it is critical to develop new anode materials for SIBs for practical use. Here, heteroatom‐doped graphene with high doping levels and disordered structures is prepared using a simple and economical thermal process. The solvothermal‐derived graphene shows excellent performance as an anode material for SIBs. It exhibits a high reversible capacity of 380 mAh g^−1^ after 300 cycles at 100 mA g^−1^, excellent rate performance 217 mAh g^−1^ at 3200 mA g^−1^, and superior cycling performance at 2.0 A g^−1^ during 1000 cycles with negligible capacity fade.

Over the past few decades, electrical energy storage has received increasing attention both in the research and industry fields, because of the rapid growth in their use in portable electronic devices and energy storage systems.^1^ Although lithium‐ion batteries (LIBs) have been regarded as the most popular power sources with many advantages such as high capacity and long cycle life, Li resources are limited and nonuniformly distributed globally. On the contrary, sodium is abundant, inexpensive, and ubiquitous.^2^ Thus, sodium‐ion batteries (SIBs) have recently attracted a great deal of attention as promising alternatives to LIBs. However, graphite, which is a commercially available anode material for LIBs, is not suitable for application in SIBs owing to the larger size of sodium ions precluding for intercalation into the interlayers of graphite.^3^

Various carbonaceous materials have been applied as potential anode materials for SIBs, including hard carbon, amorphous carbon, carbon black, activated carbon, carbon fibers, and graphene.^4^ Furthermore, different morphologies of these carbon‐based materials such as porous carbon, hollow carbon spheres, and porous carbon nanofibers have shown different electrochemical properties.^5^ These results indicate that the battery properties, especially in terms of capacity and cycle stability, could be effectively adjusted by the judicious control of physical properties, such as pore size and interlayer distance. In addition, heteroatom doping (e.g., S, P, and N) is also an efficient means to tune the chemical and physical properties of carbonaceous materials,^6^ as it can modulate electronic properties by producing defects and porosities, as well as increase the interlayer distance of carbon. Li et al. synthesized S‐doped disordered carbon that exhibited a high reversible capacity of 516 mAh g^−1^ at 20 mA g^−1^.^3^ Yang et al. prepared S‐doped N‐rich carbon nanosheets, which achieved a reversible capacity of 350 mAh g^−1^ at 50 mA g^−1^, which is much higher than N‐doped carbon.^7^ In addition, Qie et al. demonstrated that S‐doping could enlarge the interlayer distances of carbon materials. Moreover, they also claimed that S‐doped carbon was more suitable than N‐doped carbon for SIBs anodes, because doped sulfur can act as a redox site for reaction with sodium ions and enlarge interlayer distance.^8^

Recently, a gram‐scale synthetic method was developed to produce solvothermal carbon nanomaterials using liquid precursors. These carbon materials had sheet‐like 2D nanostructures.[[qv: 6a,9]] Choucair et al. first synthesized solvothermal carbon using ethanol as a source and demonstrated that solvothermal carbon can be categorized within the graphene family.^9^ Solvothermal graphene (SG) showed a higher degree of disorder than traditional graphene. Our group successfully converted S‐containing organic molecules (dimethyl sulfoxide, DMSO) into S‐doped SG (S‐SG) via a modified thermal process. Compared to traditional doping processes (e.g., chemical vapor deposition; segregation growth and post treatment, etc.), the conditions employed for this thermal process were relatively mild.^10^ In this study, the sodium storage properties of S‐SG and pristine SG have been investigated. S‐SG exhibited a high reversible capacity of 380 mAh g^−1^ after 300 cycles at 100 mA g^−1^, excellent rate performance of 217 mAh g^−1^ at 3200 mA g^−1^. In addition, it showed excellent cycling performance at 2.0 A g^−1^ during 1000 cycles with no capacity fade.

For preparation of the desired S‐SG powders, the carbon‐ and sulfur‐containing precursor (i.e., DMSO) was heated at reflux under inert conditions until formation of the desired black product was observed (**Figure** **1**a). Similarly, the pristine SG was synthesized via a modified thermal process using methanol as the carbon source. As shown in the scanning electron microscopy (SEM) image presented in Figure 1b, the as‐prepared S‐SG samples are porous and composed of nanosheets. In addition, the transmission electron microscopy (TEM) image showed that the S‐SG sample presents a thin and crumpled morphology (Figure 1c; see Figure S1 in the Supporting Information for SEM and TEM images of the pristine SG), while the high‐resolution TEM image (Figure 1d) clearly shows that the interlayer distance is significantly larger (≈0.41 nm) than that of graphite (≈0.34 nm). This expanded interlayer spacing is expected to facilitate the insertion of large Na ions between the layers.

**Figure 1:**
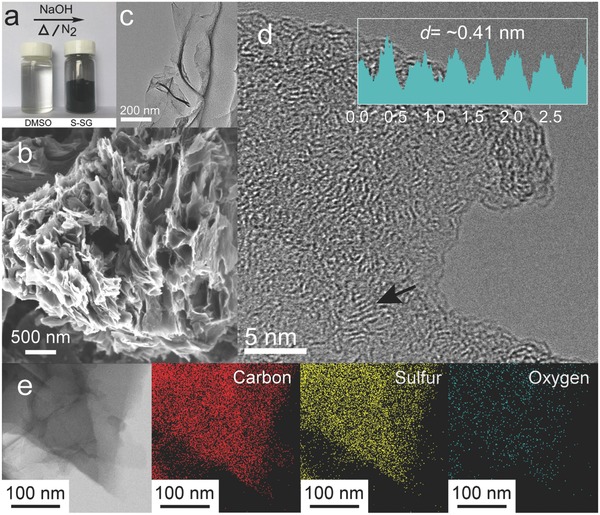
a) A photograph of DMSO and S‐SG. The transparent liquid precursor was converted to black powder through a solvothermal reaction. b) SEM, c) TEM, and d) high‐resolution TEM images of S‐SG. e) EDS elemental mapping of S‐SG.

Figure 1e presents the TEM image and corresponding energy‐dispersive X‐ray spectroscopy (EDS) mapping images of S‐SG, which show that elemental sulfur was homogeneously distributed in the S‐SG samples. Based on the elemental analysis results (Table S1, Supporting Information), the sulfur content of the S‐SG was 21.8 wt%. To better understand the chemical states of the S‐SG and pristine SG, X‐ray photoelectron spectroscopy (XPS) was carried out. In the XPS survey spectra (**Figure** **2**a), the sulfur peaks could be clearly detected in the S‐SG sample, while only carbon and oxygen were present in the pristine SG sample. It is worth noting that the peak ratio of oxygen to carbon is much lower in the S‐SG sample. Also, much smaller peaks from C—O (286.5.5 eV) and C=O bonds (288.2 eV) were observed, compared to peaks from C—C bonds (284.5 eV) in the high‐resolution C 1s XPS spectrum of S‐SG (see Figure S2, Supporting Information).[[qv: 6a]] The high‐resolution S 2p XPS spectrum of S‐SG exhibited three peaks at binding energies of 163.6, 164.8, and 168.5 eV, which correspond to S 2p_3/2_, S 2p_1/2_, and the oxidized sulfur groups, respectively (Figure 2b).[[qv: 6a,8a]] These observations suggest that the S and C atoms are covalently bonded. Furthermore, the Raman spectra of both S‐SG and pristine SG showed broad and merged disorder‐induced D‐bands and graphite G‐bands at around 1350 and 1600 cm^−1^ (Figure 2c). In the X‐ray diffraction (XRD) results (Figure 2d), broad peaks located at 22.3° and 44.3° were observed for both S‐SG and the pristine SG, corresponding to the (002) diffraction of the graphitic layered structure and the (100) diffraction of graphite, respectively.^3^, ^9^, ^11^ Broad and diffuse ring patterns are observed in selected area electron diffraction patterns of S‐SG and pristine SG (Figure S3, Supporting Information), confirming that a stacking number of carbon layers in S‐SG and pristine SG is smaller than that of graphite. This is in good agreement with XRD and Raman results.[[qv: 6f]] The interlayer distances were calculated based on Bragg's law and were found to be about 0.40 nm for both samples, thereby confirming the TEM result presented in Figure 1d. Moreover, the nitrogen adsorption and desorption isotherms and pore size distributions of the S‐SG and pristine SG were also investigated (Figure S4, Supporting Information). The adsorption isotherms are classified into several types, and the types of isotherms of S‐SG and pristine SG could be classified as type IV isotherms, indicating the presence of mesoporous structures with Brunauer‐Emmett‐Teller surface areas of 308 and 741 m^2^ g^−1^, respectively.[[qv: 6a]] The Barret–Joyner–Halenda adsorption average pore diameters of S‐SG and SG were determined to 10.4 and 9.4 nm, respectively (Figure S4b, Supporting Information).

**Figure 2:**
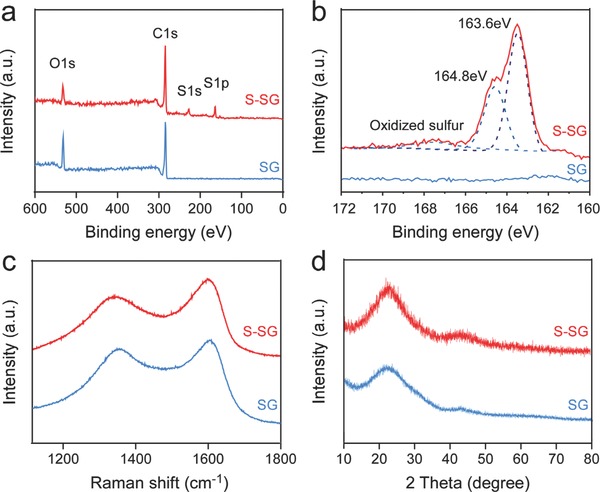
a) XPS survey spectra of S‐SG and pristine SG. b) High‐resolution S 2p XPS spectra of the S‐SG and pristine SG with S 2p_3/2_, S 2p_1/2_, and oxidized sulfur. c) Raman spectra of S‐SG and pristine SG. d) XRD patterns of S‐SG and pristine SG.

To evaluate the electrochemical properties of S‐SG and the pristine SG, coin cells were galvanostatically discharged/charged in the range of 0.01–3.0 V (vs. Na^+^/Na). **Figure** **3**a,b presents the voltage profiles of pristine SG and S‐SG electrodes during the initial 5 cycles at a current density of 100 mA g^−1^. As indicated in Figure 3a, pristine SG delivered initial discharge and charge capacities of 1045.4 and 302.4 mAh g^−1^, respectively. The initial Coulombic efficiency of pristine SG was 28.9%. On the contrary, S‐SG exhibited a significantly improved Coulombic efficiency of 55.6%. The Coulombic efficiency of the S‐SG is almost twice than that of pristine SG because S‐SG has lower surface area (Figure S4a, Supporting Information) and lower oxygen content (Table S1, Supporting Information). Also, S‐SG delivered higher sodiation/desodiation capacities of 877.8/488.0 mAh g^−1^ (Figure 3b). The high reversible capacity of over 400 mAh g^−1^ was retained during subsequent cycles for the S‐SG electrode.

**Figure 3:**
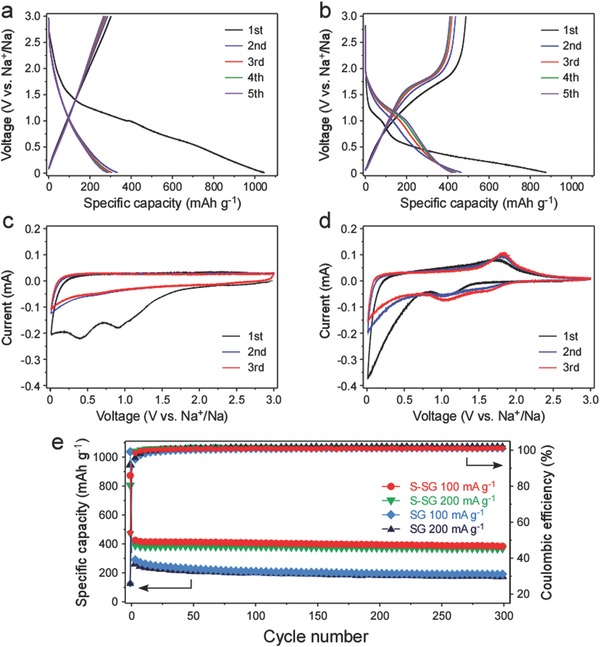
Voltage profiles of a) pristine SG and b) S‐SG at a current density of 100 mA g^−1^. Cyclic voltammograms of c) pristine SG and d) S‐SG at a scan rate of 0.1 mV s^−1^. e) Cycle performance of pristine SG and S‐SG at current densities of 100 and 200 mA g^−1^.

The initial three cyclic voltammograms (CVs), which were obtained at a scan rate of 0.1 mV s^−1^, are shown in Figure 3c,d for the pristine SG and for S‐SG, respectively. In the case of the pristine SG, two well‐defined peaks at about 1.0 and 0.4 V disappeared after first reduction. The peak observed at 1.0 V was attributed to the irreversible reaction of the functional groups with sodium ions. Another cathodic peak at 0.4 V is related to formation of solid electrolyte interphase (SEI) layer.^12^ Contrary to CVs of the pristine SG, two additional redox couples were observed at 1.6/2.2 and 1.1/1.8 V in CVs of S‐SG. These redox couples are resulted from the reaction of doped sulfur with Na ions, so these are often found in CVs of room temperature Na‐S batteries.^3^, ^13^ Reduction peak at 1.6 V is ascribed to reaction between S and Na ions to form Na_2_S_2_. The second cathodic peak at 1.1 V is related to reduction of Na_2_S_2_ to Na_2_S. It is worthwhile to note that S‐SG presents a number of advantages as an SIB anode material compared to pristine SG or other graphene with oxygen functional groups. The electrochemically active sulfur can enhance the capacity, while the reaction between S and Na is significantly more reversible than that between O and Na, in addition to exhibiting lower voltage hysteresis. Furthermore, S‐SG has a particularly high lattice spacing related to that of pristine SG, which can facilitate the insertion of Na ions into the layers, even in the presence of lower quantities of oxygen (see Table S1 in the Supporting Information for elemental analysis, also discussed in XPS section above).

The cycle performances of pristine SG and S‐SG at current densities of 100 and 200 mA g^−1^ are displayed in Figure 3e. The S‐SG delivered a reversible capacity of 379.5 mAh g^−1^ at 100 mA g^−1^ in the 300th cycle with a negligible capacity fade during 300 cycles, while the capacity of pristine SG kept decreasing to 182.8 mAh g^−1^ at the 300th cycle. The reversible capacity of S‐SG is almost twice as high as that of pristine SG, and its Coulombic efficiency is over 99.8% at 100 mA g^−1^. Interestingly, the capacity and cycle performance of both S‐SG and pristine SG at 200 mA g^−1^ are similar at 100 mA g^−1^, suggesting that the rate capabilities of both materials are excellent. The clear capacity fade during the initial 50 cycles in pristine SG, which is likely to be due to the less reversible reaction between O and Na, is rarely observed in S‐SG. Even at higher current density of 500 mA g^−1^_,_ its discharge capacity is over 300 mAh g^−1^ (Figure S5, Supporting Information).

As shown in **Figure** **4**a, the rate properties of the pristine SG and S‐SG were evaluated by varying the current density stepwise from 50 to 3200 mA g^−1^, and then back to 50 mA g^−1^. Both S‐SG and pristine SG showed high cycle stability at all current densities, including the very high current density of 3200 mA g^−1^. However, S‐SG showed much higher capacities than those of pristine SG, which were 436.0, 398.1, 365.5, 329.0, 290.3, 251.2, 217.1, and 422.6 mAh g^−1^ at 50, 100, 200, 400, 800, 1600, 3200, and 50 mA g^−1^, respectively. In particular, the capacity of S‐SG remained at over 200 mAh g^−1^ even at 3200 mA g^−1^, while the capacity of pristine SG dropped to 147.8 mAh g^−1^. The capacities of S‐SG recovered to high values when the current density decreased back to 50 from 3200 mA g^−1^, indicating that the structure of S‐SG is well‐maintained even after high rate cycling. The high rate capability of S‐SG is resulted from S‐doping, which increases electrical conductivity and facilitate the Na ion transport by expanding interlayer spacing. To further explain the slight capacity difference at same current density during the constant current cycling test (figure 3e) and stepwise current cycling test (figure 4a), the voltage profiles and corresponding differential capacities were plotted in Figure S6 (Supporting Information). As can be seen in Figure S6 (Supporting Information), the reaction voltage shifted during the initial cycles, which is attributed to activation process of the reaction between doped sulfur and Na ions. During this process, the total capacity also slightly changes, and this change is more dramatic in the lower current density (Figures S6a,b and Figure S7, Supporting Information). Therefore, the capacities at same rate in different cycling sequences (constant current cycling test, and stepwise current cycling test) can be slightly different due to the different degree of activation occurs in two different cycling).

**Figure 4:**
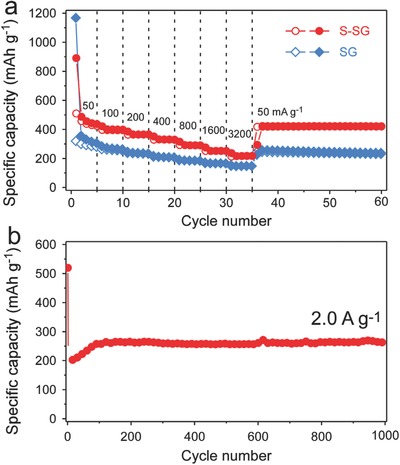
a) Rate properties of S‐SG and pristine SG at various current densities. b) Cycle performance of S‐SG at a current density of 2.0 A g^−1^.

The cycle performance of S‐SG at 2.0 A g^−1^ is shown in Figure 4b. As indicated, after the initial cycle, the specific capacity increased continuously during the initial cycles, especially at high current densities, as is often observed for carbonaceous materials.[[qv: 3,8b,14]] Once the capacity is stabilized after about 100 cycles (>250 mAh g^−1^), it was maintained for 900 cycles without capacity fade. The electrochemical properties of S‐SG and the pristine SG were compared with those of other reported materials in Table S2 (Supporting Information), including the carbon materials and metal oxides.^15^ Notably, S‐SG exhibited the highest electrochemical performance among them. In addition, pristine SG also exhibited relatively high cycle stability for significant periods, when compared to other undoped carbon materials, showing that solvothermal method is a superior way to prepare graphene for SIB anodes. The main reasons for the excellent cycle performance of SG are likely due to the large interlayer distances, highly disordered structures and greater number of active sites for Na ion storage than others. In addition, by doping with sulfur, as well as lowering the oxygen content, the capacity and cycle performance of S‐SG were significantly enhanced.

Electrochemical impedance spectroscopy was performed before and after the 1st cycle for pristine SG and S‐SG. As shown in **Figure** **5**a, each cells exhibit only one semicircle at high frequencies before cycling. The semicircle of S‐SG is much smaller than that of pristine SG before cycling, indicating that the charge transfer resistance of S‐SG (*R*_ct_: 282.3 Ω) decreased with surface area decrease by high doping level of sulfur. After the 1st cycle, two semicircles were showed in high and medium frequencies indicating that the formation of the SEI film. As can be seen in Figure 5b, both samples' semicircles are decreased. From the fitted results, obtained according to the equivalent circuit shown in the inset of Figure 5b, both the charge transfer resistance and the SEI film resistance of S‐SG (*R*_ct_: 154.9 Ω, *R*_SEI_: 14.2 Ω) were much lower than those of the pristine SG (*R*_ct_: 281.1 Ω, *R*_SEI_: 93.8 Ω), demonstrating that S‐doping in SG decreases both the SEI layer resistance (*R*_SEI_) and charge transfer resistance (*R*_ct_).

**Figure 5:**
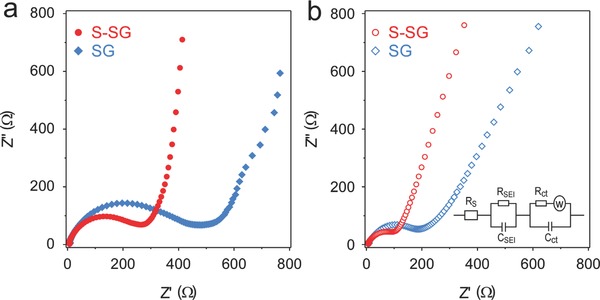
Electrochemical impedance spectra for S‐SG and pristine SG a) before cycling, and b) after 1 cycle.

In Summary, solvothermal‐derived sulfur‐doped graphene (S‐SG) was synthesized via a simple, economical, one‐pot method as an anode electrode material for SIBs. We found that the prepared S‐SG exhibited a highly disordered structure, a large interlayer distance, and large numbers of active sites. As an anode for SIB, S‐SG exhibited a high reversible capacity of 380 mAh g^−1^ after 300 cycles at 100 mA g^−1^, an excellent rate performance of 217 mAh g^−1^ at a particularly high current density of 3200 mA g^−1^, and a superior cycling performance of 263 mAh g^−1^ at 2.0 A g^−1^ over 1000 cycles. To the best of our knowledge, S‐SG exhibited the best electrochemical performance of other previously reported S‐doped carbon materials. For comparison, pristine SG was also prepared and exhibited a high performance of 182.8 mAh g^−1^ at 100 mA g^−1^ after 300 cycles. It could be concluded that solvothermal‐derived carbon materials with high levels of sulfur doping and a highly disordered structure are suitable for use as anode materials for high‐performance SIBs. Indeed, we expect that such solvothermal‐based carbon materials can be further extended to other energy‐related applications.

## Experimental Section

*Reagents*: DMSO (99.7%) was purchased from Acros Organics (NJ, USA). Hydrochloric acid (35%–37%), sodium metal, sodium hydroxide (NaOH, 98%), and methanol (99%) were purchased from Samchun Pure Chemical (Seoul, South Korea). Deionized water was filtered using a water purification system. Unless otherwise stated, all chemicals were used as received without further purification.

*Synthesis of S‐SG*: S‐SG was synthesized according to a previously reported method.[[qv: 6a]] Briefly, a mixture of DMSO (20 mL) and NaOH (4 g) was added into a flask and heated at 300 °C under a flow of nitrogen gas. Under these reflux conditions, the colorless liquid turned dark and resulting in black cake‐like substances. These black samples were washed with deionized water several times, and dried in a vacuum oven at 70 °C.

*Synthesis of Pristine SG*: Pristine SG was also synthesized according to a previously reported literature method.[[qv: 6a]] 15 g of sodium metal and 50 mL of methanol were charged to a Teflon‐lined autoclave, which was then sealed and heated at 190 °C for 36 h. After the autoclave was cooled down to room temperature, the resulting solid was annealed at 500 °C under a flow of nitrogen gas. The product was washed with a 10 wt% aqueous HCl solution and deionized water several times. Caution: The sodium metal‐containing solid sample can easily catch fire, when exposed to air.

*Characterization*: Field‐emission SEM (SUPRA 55VP) and TEM (JEM‐2100F) coupled with EDS (Oxford Instrument) were employed to examine the morphology and composition of the S‐SG and pristine SG samples. The crystal structures were determined by XRD (Bruker D‐5005) using Cu Ka radiation at λ = 1.54 Å. The Raman spectra were obtained using a Dongwoo DM500i Raman spectrometer. XPS was carried out on a Sigma Probe instrument (ThermoFisher Scientific) with Al Kα (1486.8 eV) as the X‐ray source. Elemental analysis was carried out with a Thermo Scientific Flash 2000 organic elemental analyzer installed at the National Center for Inter‐university Research Facilities (NCIRF) at Seoul National University. Nitrogen adsorption and desorption isotherms were obtained using a Micromeritics ASAP 2020 surface area and porosity analyzer.

*Electrochemical Characterization*: Electrodes were made by mixing the 70 wt% of active material, 15 wt% of super P, and 15 wt% of poly (acrylic acid) in *n*‐methyl‐2‐pyrrolidinone. Afterward, the slurries were dried in a vacuum oven overnight. Sodium metal was used as the counter electrode, while a glass fiber GF/C (Whatman) and 1.0 m NaClO_4_ in ethylene carbonate/propylene carbonate (1:1 by volume) were used as the separator and the electrolyte, respectively. In addition, 5 wt% fluoroethylene carbonate was added to the electrolyte as an additive. The loading levels of the active materials were 1 mg cm^−2^. 2032‐type coin cells were assembled in an Ar‐filled glove box and tested using a WBCS3000 cycler (WanATech) in the range from 0.01 to 3.0 V (vs. Na^+^/Na) at 25 °C.

## Conflict of Interest

The authors declare no conflict of interest.

## Supporting information

Supplementary
