# Supplementary material for: Solvothermal‐Derived S‐Doped Graphene as an Anode Material for Sodium‐Ion Batteries
Source: Adv Sci (Weinh). 2018 Feb 14;5(5):1700880. doi: 10.1002/advs.201700880 (PMC5979751; doi:10.1002/advs.201700880)
Supplement: Supplementary file 1 — Supplementary [file ADVS-5-1700880-s001.pdf]

Copyright WILEY-VCH Verlag GmbH & Co. KGaA, 69469 Weinheim, Germany, 2018.

ADVANCED SCIENCE
Open Access

Supporting Information

for *Adv. Sci.,* DOI: 10.1002/advs.201700880

Solvothermal-Derived S-Doped Graphene as an Anode Material for Sodium-Ion Batteries

Bo Quan, Aihua Jin, Seung-Ho Yu, Seok Mun Kang, Juwon Jeong, Héctor D. Abruña, Longyi Jin, Yuanzhe Piao, and Yung-Eun Sung**

Copyright WILEY-VCH Verlag GmbH & Co. KGaA, 69469 Weinheim, Germany, 2016.

Supporting Information

Solvothermal-Derived S-doped Graphene as an Anode Material for Sodium-Ion Batteries

Bo Quan, Aihua Jin, Seung-Ho Yu, Seok Mun Kang, Juwon Jeong, Héctor D. Abruña, Longyi Jin, Yuanzhe Piao, Yung-Eun Sung**

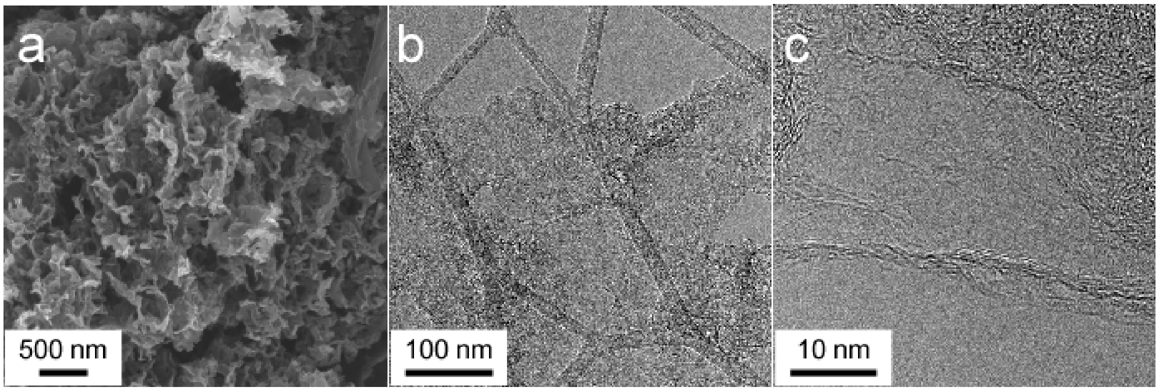

Figure S1. (a) SEM, (b) TEM and (c) high-resolution TEM images of pristine SG.

Table S1. Elemental analysis data for S-SG and pristine SG.

Sample	% Composition/Mass				
	C	H	N	S	O
**S-doped solvothermal graphene**	71.77	1.24	0.06	21.80	4.93
**Pristine solvothermal graphene**	88.83	1.50	-	-	9.48

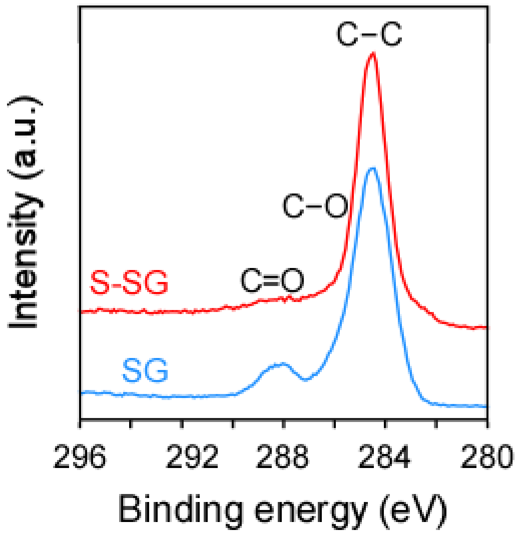

Figure S2. High resolution C1s XPS spectra of S-SG and pristine SG.

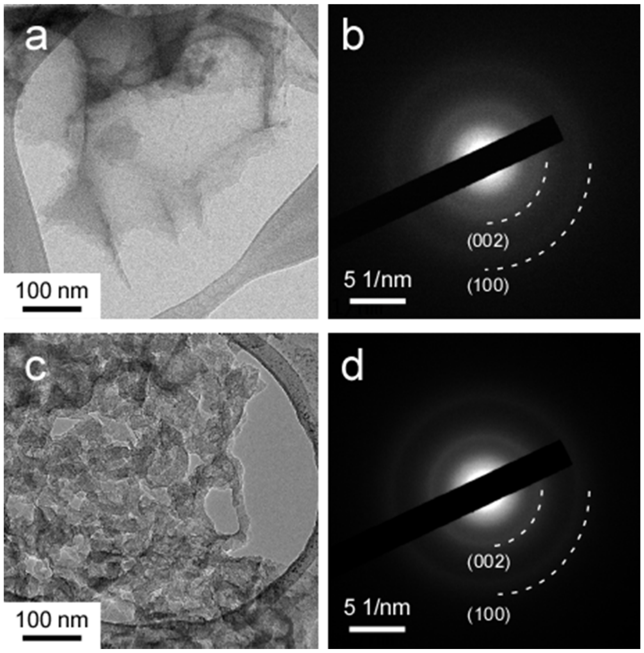

Figure S3. The TEM images (a) S-SG and (c) pristine SG, and (b, d) corresponding SAED patterns, respectively.

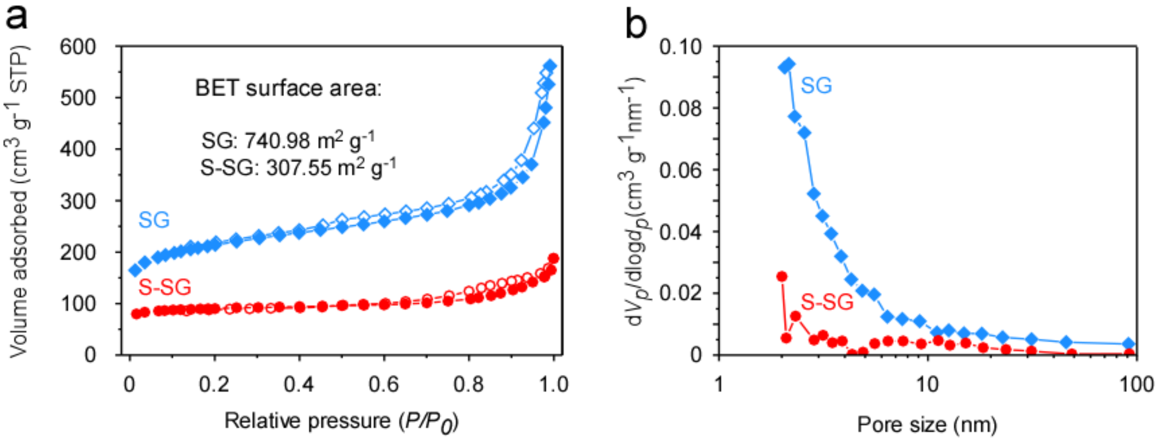

Figure S4. (a) Nitrogen-adsorption/desorption isotherms of S-SG and pristine SG. (b) BJH pore size distributions of S-SG and pristine SG as calculated from the adsorption isotherms.

Table S2. Electrochemical performances of various carbon-based materials as sodium-ion battery anode materials.

Samples	Current density mA g^{-1}	Initial discharge capacity mAh g^{-1}	Reversible capacity mAh g^{-1} (at the cycle no.)	Ref.
S-doped disordered carbon	20	887	271 (1000th cycle)	S[3]
N/O-doped carbon nanobubbles	100	~520	120 (30th cycle)	S[6c]
N-doped carbon nanotube	50	378.3	175.5 (300th cycle)	S[6d]
N-doped porous carbon sphere	200	635.8	206 (600th cycle)	S[6e]
N-doped 3D graphene foams	500	852.6	594 (80th cycle)	S[6f]
N/P-doped carbon microspheres	100	742	~200 (90th cycle)	S[6g]
S/N-doped carbon nanosheets	50	954	350 (100th cycle)	S[7]
S-doped carbon	500	640	303.2 (700th cycle)	S[8a]
S-doped flexible graphene films	100	676	244 (300th cycle)	S[8b]
Reduced graphene oxide (RGO)	40	~780	174.3 (250th cycle)	S[11]
Co_3O_4/CNTs	50	~550	403 (100th cycle)	S[15a]
MoS_2/graphene	20	~1700	~400 (100th cycle)	S[15b]
SnO_2/graphene	20	1942.0	~700 (100th cycle)	S[15c]
SG	100	1045.4	182.8 (300th cycle)	This work
S-SG	100	877.7	380.0 (300th cycle)	This work

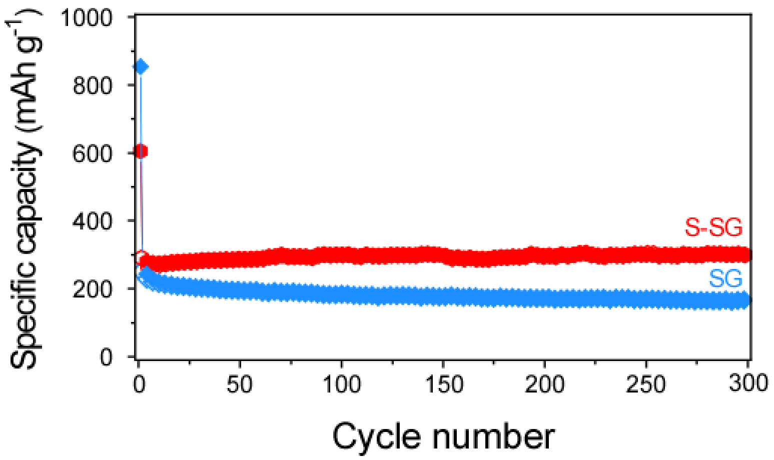

Figure S5. Cycle performance of S-SG and pristine SG at a current density of 500 mA g^{-1}.

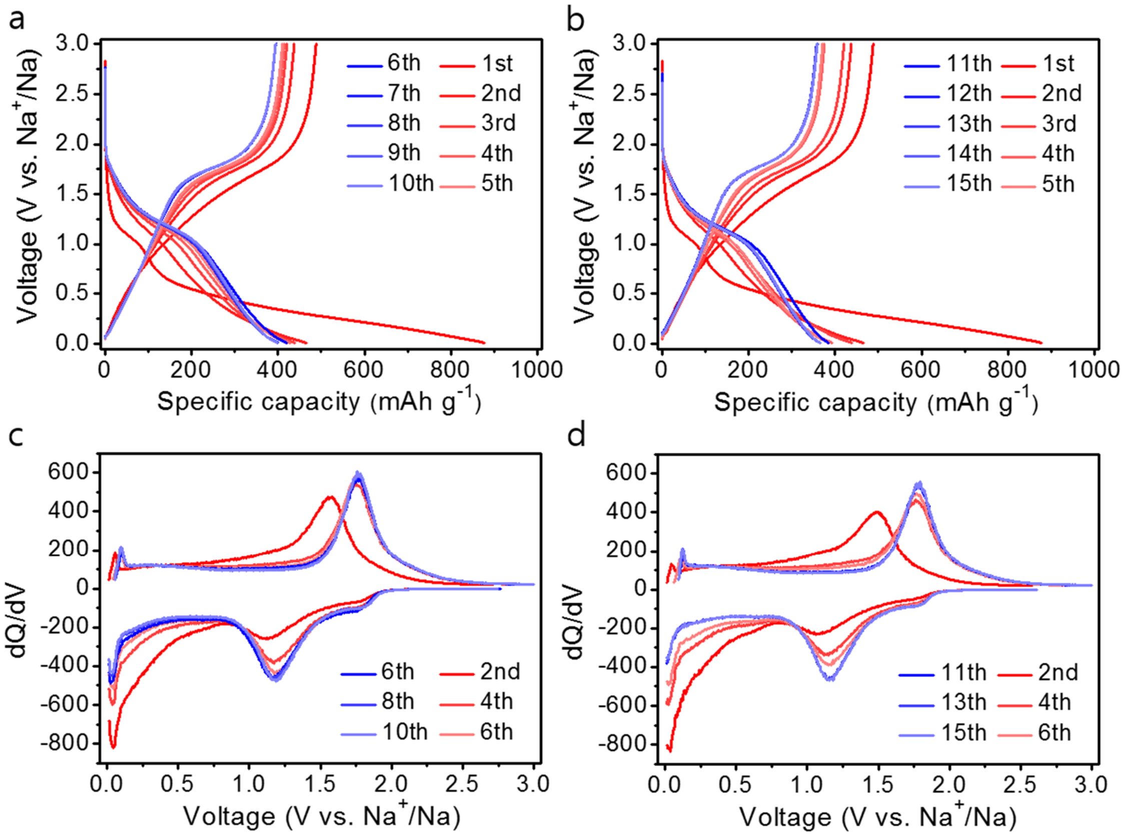

Figure S6. The voltage profiles of S-SG at a current density of (a) 100 mAh g^{-1} (red: from constant current cycling, blue: from stepwise current cycling) and (b) 200 mAh g^{-1} (red: from constant current cycling, blue: from stepwise current cycling); the differential capacity plots of S-SG at a current density of (c) 100 mAh g^{-1} (red: from constant current cycling, blue: from stepwise current cycling) and (d) 200 mAh g^{-1} (red: from constant current cycling, blue: from stepwise current cycling).

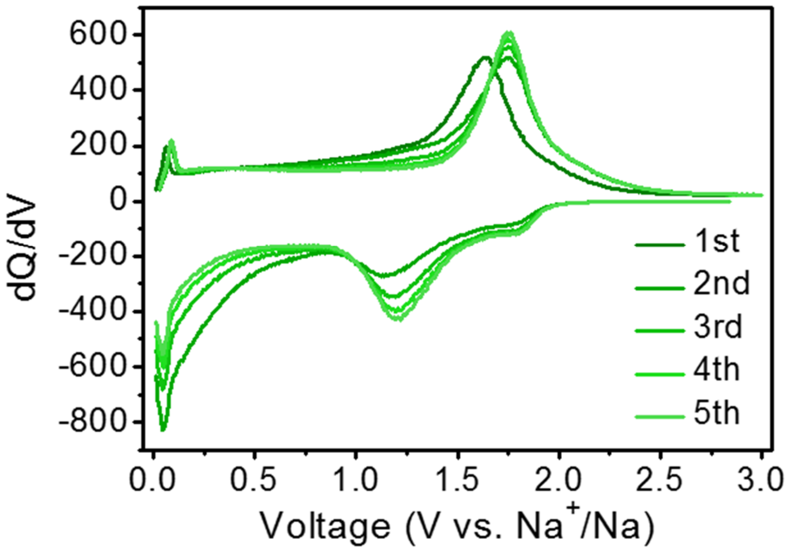

Figure S7. The differential capacity plots of S-SG at a current density of 50 mAh g^{-1}.
